# Development of 20(S)-Protopanaxadiol-Loaded SNEDDS Preconcentrate Using Comprehensive Phase Diagram for the Enhanced Dissolution and Oral Bioavailability

**DOI:** 10.3390/pharmaceutics12040362

**Published:** 2020-04-15

**Authors:** Young Hoon Kim, Yu Chul Kim, Dong-Jin Jang, Kyoung Ah Min, Jenisha Karmacharya, Thi-Thao-Linh Nguyen, Han-Joo Maeng, Kwan Hyung Cho

**Affiliations:** 1 College of Pharmacy and Inje Institute of Pharmaceutical Sciences and Research, Inje University, Gimhae 50834, Korea; net5855@naver.com (Y.H.K.); minkahh@inje.ac.kr (K.A.M.); 2 Department of Pharmaceutical Engineering, Inje University, Gimhae 50834, Korea; yckim@inje.ac.kr (Y.C.K.); djjang@inje.ac.kr (D.-J.J.); 3 College of Pharmacy, Gachon University, Incheon 21936, Korea; karmacharya.jenisha@gmail.com (J.K.); linhnguyen@gachon.ac.kr (T.-T.-L.N.)

**Keywords:** 20(S)-protopanaxadiol, self-nanoemulsifying drug delivery system, comprehensive ternary phase diagram, oral bioavailability

## Abstract

In this study, we aimed to develop a 20(S)-protopanaxadiol (PPD)-loaded self-nanoemulsifying drug delivery system (SNEDDS) preconcentrate (PSP) using comprehensive ternary phase diagrams for enhanced solubility, physical stability, dissolution, and bioavailability. Capmul MCM C8 and Capryol 90 were selected as the oil phase owing to the high solubility of PPD in these vehicles (>15%, w/w). Novel comprehensive ternary phase diagrams composed of selected oil, surfactant, and PPD were constructed, and the solubility of PPD and particle size of vehicle was indicated on them for the effective determination of PSP. PSPs were confirmed via particle size distribution, physical stability, and scanning electron microscope (SEM) with the dispersion of water. The optimized PSP (CAPRYOL90/Kolliphor EL/PPD = 54/36/10, weight%) obtained from the six possible comprehensive ternary phase diagrams showed a uniform nanoemulsion with the particle size of 125.07 ± 12.56 nm without any PPD precipitation. The PSP showed a dissolution rate of 94.69 ± 2.51% in 60 min at pH 1.2, whereas raw PPD showed negligible dissolution. In oral pharmacokinetic studies, the PSP group showed significantly higher C_max_ and AUC_inf_ values (by 1.94- and 1.81-fold, respectively) than the raw PPD group (*p* < 0.05). In conclusion, the PSP formulation with outstanding solubilization, dissolution, and in-vivo oral bioavailability could be suggested using effective and comprehensive ternary phase diagrams.

## 1. Introduction

20(S)-Protopanaxadiol (PPD), a fully deglycosylated active metabolite in the gastrointestinal tract derived from various ginsenosides such as Rb1, Rb2, Rb3, Rh2, Rg3, Rg2, and compound K, has drawn attention because of its diverse and valuable pharmacological activities [1,2,3]. PPD has been known to exert antitumor, antimetastasis, antiestrogen, anti-inflammatory, cardioprotective, antidepression, and anti-photoaging (i.e., antiwrinkle and skin-whitening) effects [4,5]. Its various therapeutic effects are considered to be mediated via stimulation of the central nervous system, induction of apoptosis, and protection from DNA injury or chemoprophylaxis [6,7,8,9,10,11]. It has been reported that PPD possesses similar or stronger pharmacological activities than representative ginsenosides, Rh2 and Rg3 [12]. PPD, an active metabolite form itself, exerts various therapeutic benefits upon administration via oral or topical routes. However, PPD has extremely poor water solubility (<50 ng/mL), which limits its oral absorption [13]. Therefore, to enhance the oral bioavailability of PPD, its aqueous solubility should be improved by preparing oral formulations using an appropriate solubilization technology.

**Figure 1:**
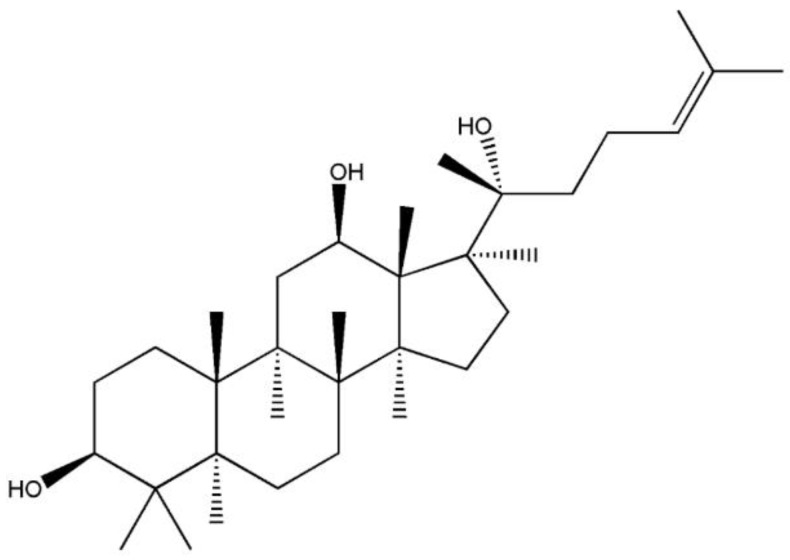
Structure of 20(S)-Protopanaxadiol.

To improve the dissolution and in-vivo oral bioavailability of PPD, researchers have utilized several formulation technologies, such as nanosuspension formulations prepared by anti-solvent precipitation methods or solid dispersions by melting solvent methods [12,14]. However, these methods had several disadvantages, including complicated preparation processes, low yield, and limited bioavailability. The structure of PPD comprises a tetracyclic terpene sapogenin containing hydrocarbon rings, which makes this molecule water-insoluble and fairly lipophilic (Figure 1). As a solubilization technology, the self-nanoemulsifying drug delivery system (SNEDDS) has been widely applied to enhance the aqueous solubility of highly lipophilic drugs such as PPD [15,16,17,18,19]. SNEDDS formulations are generally composed of oils, surfactants (or co-surfactants), and water, and they increase drug solubilization by forming thermodynamically stable colloidal nanoemulsions in aqueous media with a particle size range of 50–200 nm [20,21]. Drug molecules are incorporated into the interfacial film layer or oil core droplets, which have a large total surface area. Thus, SNEDDS can enhance drug solubility and gastrointestinal absorption, thereby improving oral bioavailability [15].

In general, the optimized compositions of SNEDDS can be determined by typical pseudo-ternary phase diagrams constructed using oil, surfactant/co-surfactant, and water. The phase diagram is an effective tool to determine vehicle compositions, providing stable particulate dispersions, such as uniformly sized nanoemulsions. However, the solubilization degree of drugs, which affects the dispersion of SNEDDS, is difficult to comprehensively confirm using the conventional phase diagram. Thus, a more effective method using a modified phase diagram is recommended to reflect the effects of drug contents on the formulation and all other ingredients to obtain the optimal formulation.

Basically, a SNEDDS preconcentrate refers to the state of the formulation before water is added to form a dispersion. If a SNEDDS preconcentrate is spontaneously dispersed and forms a nanoemulsion in a sufficient amount of water, it can be a good formulation by itself. A phase diagram constructed using a combination of drug and vehicle components, such as a surfactant and/or co-surfactant, and oil may contribute to determining the composition of a candidate SNEDDS preconcentrate with desirable properties. Drug loading content (%) and the optimized region for a SNEDDS preconcentrate can be indicated using a phase diagram. The solubility of the drug in each oil or surfactant type can be incorporated to modify the phase diagram and ultimately enable a more comprehensive approach for suggesting drug-soluble compositions.

The present study suggested that the formulations of PPD-loaded SNEDDS preconcentrate (PSP) may be efficiently optimized by a novel comprehensive phase diagram method based on physicochemical examination. The regions in the phase diagram present the factors determining the formation of SNEDDS preconcentrates (i.e., vehicle dispersibility, drug loading content (%), and drug solubility). The optimized PSP formulations were evaluated in a dissolution study and an in-vivo oral pharmacokinetics study. The construction of comprehensive phase diagrams suggested in this study could be a promising strategy for developing oral formulations containing drug molecules with the challenging characteristics of low solubility and poor oral bioavailability.

## 2. Materials and Methods

### 2.1. Materials

PPD (purity >99.9%, Figure 1) was purchased from Chengdu Biopurify Phytochemicals, Ltd. (Chengdu, China). Kolliphor HS15 (polyoxyl 15 hydroxystearate), Kolliphor EL (polyoxyl castor oil), Kolliphor PS 80 (polysorbate 80), and Kollisolv MCT 70 (medium chain triglycerides) were provided by BASF (Ludwigshafen, Germany). Capmul MCM C8 (mono/diglycerides of caprylic acid) and Capryol 90 (propylene glycol monocaprylate (type II) were provided by Gattefosse (Saint-Priest, France). All other chemicals were of reagent grade and were used without further purification.

### 2.2. HPLC Analysis

The PPD content in samples was quantified using a Waters 2695 HPLC system (Waters, Milford, MA, USA) equipped with a UV–Vis detector (Waters 2487; Waters, Milford, MA, USA). PPD was separated on a reverse-phase column (C18, 5 µm, 4.5 mm × 25 cm) (Shiseido, Tokyo, Japan) at a flow rate of 1.2 mL/min. The mobile phase comprised acetonitrile and water (95:5, v/v). The injected volume of the sample was 10 µL, and UV detection was performed at 203 nm. Data acquisition and processing were carried out using the Waters LC Solution software.

### 2.3. Solubility Test

The solubility of PPD was evaluated in various oils (Capmul MCM C8, Capryol 90, or Kollisolv MCT 70) and surfactants (Kolliphor EL, Kolliphor PS80, or Kolliphor HS15). An excess amount of PPD powder (approximately 50 mg) was added to each type of oil or surfactant. After stirring in a water bath at 30 or 50 °C for 40 min, the mixture was centrifuged at 15,000 rpm for 10 min. The supernatant was diluted with the mobile phase used in the HPLC procedure. The amount of PPD was measured in triplicate by HPLC.

### 2.4. Particle Size Measurement

After 50 mg of each vehicle and formulation was dispersed in 3 mL of 37 °C water by vortex-mixing for 1 min, the particle size was measured using a dynamic light scattering (DLS) instrument (NanoBrook 90Plus; Brookhaven instruments Co., Holtsville, NY, USA). The results of triplicate measurements were averaged and are depicted in plots.

### 2.5. Preparation and Physical Characterization

Vehicles were prepared by mixing each oil and surfactant component from two oils and three surfactants at five different weight ratios (oil:surfactant = 8:2, 6:4, 5:5, 4:6, or 2:8) as selected in the above solubility test (Section 2.3). The particle size of the vehicles after dispersion in water was measured in triplicate, and phase separation was visually observed.

### 2.6. Construction of Comprehensive Ternary Phase Diagram

Ternary phase diagrams were constructed for the vehicles with six different combinations of two oils and three surfactants. PPD solubility in each vehicle system constructed with the different ratios of oil and surfactant was calculated using Equation (1) and presented as the E value. Next, each E value was plotted on the ternary phase diagram, along with the calculated composition (%) of oil (C′) and surfactant (D′) in the vehicle system [15]. In Equation (1), the combination ratios of C and D were predetermined in the range of 100:0 ~ 0:100 (C:D, %) with a 5% interval value. Two solubility curves at 30 °C and 50 °C were drawn on the phase diagram and the solubilization region of PPD could be defined according to the temperatures.

PPD solubility (E, %) at the mixture of C and D = [(A × C) + (B × D)]/100(1)

**Figure 2:**
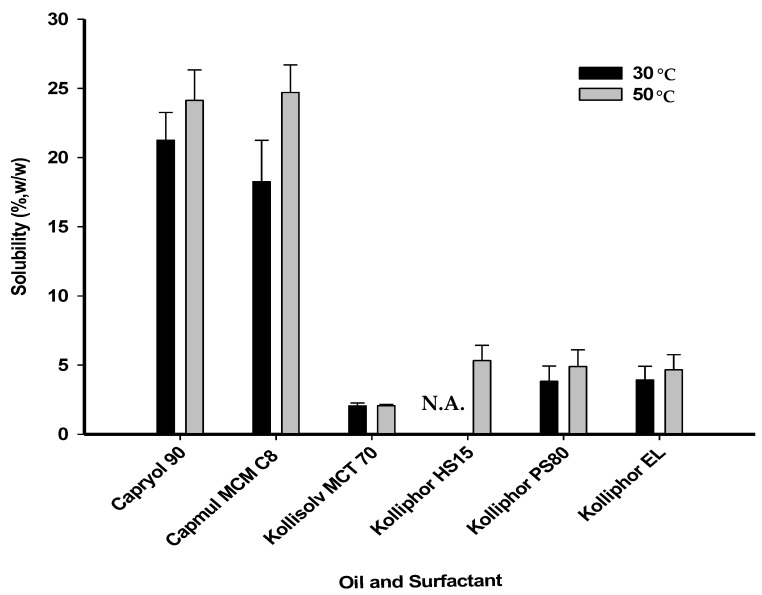
Solubility of PPD in various oils and surfactants. N.A. is not applicable to a temperature of 30 °C, as the melting point of Kolliphor HS15 (over 30 °C) was considered.

A = PPD solubility (%) in oil from Figure 2
B = PPD solubility (%) in surfactant from Figure 2
C = predetermined composition (%) of oil
D = predetermined composition (%) of surfactant
C + D = 100%
C′ = composition (%) of oil at the calculated E point
D′ = composition (%) of surfactant at the calculated E point
C′ + D′ + E = 100%

Vehicle dispersion systems without PPD were categorized into three classes (p.s. < 200 nm, 200 nm < p.s. < 300 nm, and 300 nm < p.s.) depending on the mean particle size values (abbreviated as p.s.). This was indicated on the side line displaying the only combination of oil or surfactant using symbols representing particle size classes. The dotted inner line was drawn from the symbol of the vehicle to the counter vertex representing the PPD content of 100%. All the compositions on this dotted inner line contained oil and surfactant at a fixed ratio same with the starting vehicle composition. The PPD formulations with the highest PPD loading (%) were selected from the compositions belong to the soluble region and evaluated in terms of dispersibility in an aqueous medium and particle size.

### 2.7. Observation of Precipitation

PSP formulations with high solubilization within each ternary phase diagram were selected for the stability test. For this test, 50 mg of each formulation was dispersed in 1.5 mL of 37 °C water by slight vortex mixing for 1 min. Next, the dispersion was left at room temperature for 2 h and centrifuged at 15,000 rpm for 5 min. Subsequently, it was visually checked for precipitation.

### 2.8. Scanning Electron Microscopy (SEM) Analysis

Representative morphology and size of raw PPD and the PPD-loaded formulations were analyzed using a field emission scanning electron microscope (FE-SEM; S-4300SE; Hitachi, Ltd., Tokyo, Japan). Each sample dilution was dispensed dropwise onto a TEM grid (01813-F; Ted Pella, Inc., CA, USA) and dried for 24 h. The prepared sample was placed on a carbon tape and vacuum coated. All measurements were conducted at ambient temperature.

### 2.9. Dissolution Test

Dissolution tests of raw PPD and the PSP formulations were performed by the paddle method using a VK 7000 dissolution tester (VanKel, Cary, NC, USA). Each sample equivalent to 50 mg of PPD was filled into a gelatin capsule shell and placed into a dissolution tester with a sinker. The dissolution test was performed under conditions of 50 rpm paddle speed in 900 mL of a pH 1.2 buffer at 37 ± 0.5 °C. A 5-mL aliquot of the samples was collected at a predetermined time interval and filtered through a 0.45-µm membrane filter (DISMIC^®^-13HP; ADVANTEC^®^, Tokyo, Japan). The amount of PPD in the filtrate was determined by the HPLC method described above.

### 2.10. Oral Pharmacokinetic Study in Rats

In-vivo oral pharmacokinetic studies were performed to compare the extent of oral absorption between raw PPD and the optimized PSP formulation in male Sprague Dawley rats. All animal experiments were performed according to the Guidelines for Animal Care and Use issued by Gachon University. The animal experimental protocol used in the present study was reviewed and approved by the Animal Care and Use Committee of Gachon University (#GIACUC-R2019024, approved on 14 August 2019). Sprague Dawley rats (body weight, 260–280 g) were obtained from Orient Bio (Seongnam, Korea). Before the pharmacokinetic study, the rats were fasted overnight and allowed free access to water. For blood collection, rats were anesthetized using a intramuscular injection of Zoletil (20 mg/kg) and the femoral artery was cannulated with a polyethylene tube (PE50; Clay Adams, Parsippany, NJ, USA) filled with 20 IU/mL heparinized saline, as described previously [15,22]. After recovery from surgery, the rats were administered a single oral dose (10 mg/kg) of raw PPD or optimized PSP (#F9). Next, approximately 100 μL of blood was withdrawn at predetermined time intervals: 0 (blank), 15, 30, 60, 90, 120, 240, 360, 480, and 1440 min. After the blood samples were centrifuged at 12,000 g at 4 °C, the supernatant was collected and stored at −20 °C until analysis. Plasma samples were denatured by adding a two-fold volume of methanol containing 500 ng/mL of an internal standard (IS), ketoprofen. Upon quick vortex mixing, the samples were centrifuged at 12,000 g at 4 °C. Then, the supernatant was injected into the LC-MS/MS system. The LC-MS/MS system consisted of an AB SCIEX Triple Quad 5500 (Applied Biosystems-SCIEX, Concord, Canada) equipped with a turbo ion spray interface in positive ionization mode, an Agilent LC 1200 Binary pump system (Agilent Technologies, Santa Clara, CA, USA), and a CTC analytics autosampler (CTC Analytics AG, Zwingen, Switzerland). The drug and internal standard (IS) were separated from endogenous substances in the plasma using a Synergy 4 μm polar-RP 80A column (7.5 mm × 2.0 mm, 2.6 μm; Phenomenex, Torrance, CA, USA). The mobile phase was a mixture of acetonitrile and 0.1% formic acid (80:20) at a flow rate of 0.2 mL/min. The retention time of PPD and IS were 2.3 min and 1.5 min, respectively. Multiple reaction monitoring transition in the ESI positive mode was conducted under the following conditions: PPD, *m/z* 461.4 → 424.5; IS, *m/z* 255.1 → 208.2. Mass data were acquired using Analyst software (version 1.5.2; Applied Biosystems-SCIEX, Concord, Canada). The dynamic range was 2 to 10,000 ng/mL with good linearity (R = 0.9987 with the weighting of 1/x). The lower limit of quantitation was observed to be 2 ng/mL.

For the calculation of pharmacokinetic parameters, non-compartmental analysis was performed using WinNonlin 5.0.1 (Pharsight, Cary, NC, USA). The analyzed parameters included the maximum plasma concentration (C_max_), time to reach the maximum plasma concentration (T_max_), half-life (T_1/2_), area under the plasma concentration–time curve to the last time point or infinity time (AUC_last_ or AUC_inf_), and mean residence time (MRT). Relative bioavailability (BA) was calculated as the ratio of the AUC_inf_ of the optimized PSP to that of raw PPD. Significant differences between two means for unpaired data were determined using the *t*-test. All data are expressed as the mean ± standard deviation (SD).

## 3. Results

### 3.1. PPD Solubility in Various Oils and Surfactants

To determine the oil and surfactant components suitable for solubilizing PPD in the PSP, PPD solubility was evaluated in various types of oils and surfactants (Figure 2). The solubility test was performed at different temperatures, 30 °C and 50 °C. These temperature conditions facilitated the melting of a surfactant that exists in a semi-solid state at room temperature or its solubilization with decreased viscosity. As a result, the solubility of PPD at 50 °C was slightly higher than that at 30 °C, but the difference was not significant. PPD solubility in Capryol 90 or Capmul MCM C8 at 50 °C was 24.13 ± 2.20% or 24.70 ± 2.00% (w/w), respectively, which was approximately 12-fold higher than that in MCT 70 (2.05 ± 0.20%). Thus, Capryol 90 and Capmul MCM C8 were selected as the oil components for the preparation of the vehicles. In contrast, the solubility of PPD did not differ significantly among the surfactants. The solubility of PPD in Kolliphor EL, Kolliphor PS80, or Kolliphor HS15 at 50 °C was 4.65 ± 1.10%, 4.90 ± 1.20%, or 5.33 ± 1.10% (w/w), respectively. Therefore, all the surfactants tested were used in combination with the two selected oil-types (Capryol 90 and Capmul MCM C8) to prepare the vehicles.

### 3.2. Physical Characterization of Vehicles of Various Compositions

Vehicle without PPD were prepared by mixing the selected oil and surfactant at five different ratios (oil:surfactant = 8:2, 6:4, 5:5, 4:6, or 2:8, w:w). These were then used for the determination of particle size or dispersibility in water. Particle size tended to decrease as the ratio of oil to surfactant decreased in most vehicle compositions (Table 1). When Capmul MCM C8 was used with Kolliphor EL or Kolliphor HS15 at a ratio of 2:8, a clear solution was obtained in the vehicle system and particle size was not detected. Phase separation was observed when the proportion of oil was higher than that of the surfactant in the system, except for the combination of Capryol 90 and Kolliphor EL (at a ratio of 6:4), which had the smallest particle size (91.63 ± 1.45 nm) and no phase separation.

### 3.3. Construction of Comprehensive Ternary Phase Diagrams and Characterization of Formulations

**Figure 3:**
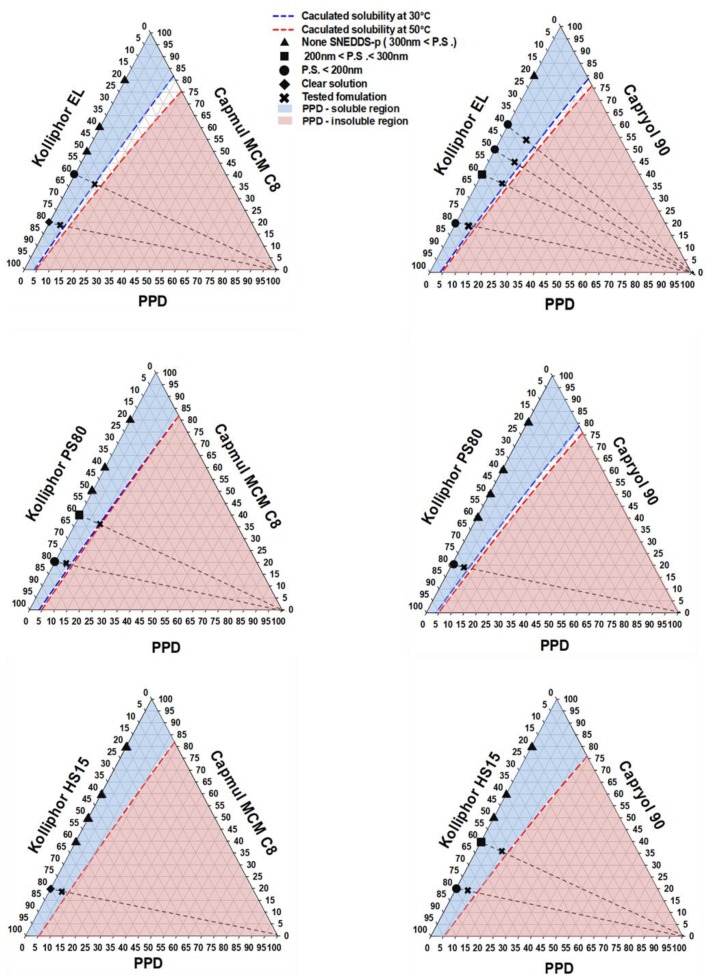
Selection of PSP formulations using comprehensive ternary phase diagrams; None SNEDDS-p, none self-nanoemulsifying drug delivery system preconcentrate; P.S., particle size.

**Figure 4:**
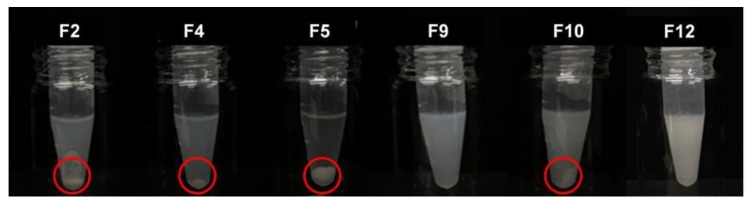
Visual observation of PSP formulations dispersed in water. Open circles indicate PPD precipitation.

Comprehensive ternary phase diagrams are shown in Figure 3, along with the corresponding calculated solubility curve of PPD in the vehicles of various compositions. The PPD-soluble region obtained from the calculated solubility curve was wider in Capryol 90 than in Capmul MCM C8. The combination of Capryol 90 as an oil and Kolliphor EL as a surfactant gave more PSP formulations than the other five combination. According to the phase diagram, a total of 12 kinds of formulations (F1~F12) were commonly liquid state at room temperature and selected based on the particle sizes of the vehicle dispersions in the soluble region. Basically, stable vehicle compositions with a particle size less than 300 nm were used for the preparation of PSP formulation. However, precipitation was observed in all formulations except for F3 (Capmul MCM C8/Kolliphor PS80/PPD = 19/76/5, weight %), F9 (Caryol 90/Kolliphor EL/PPD = 54/36/10, weight %), and F12 (Caryol 90/Kolliphor EL/PPD = 54/36/10, weight %) when dispersed in water (Table 2, Figure 4). The particle size of F3, F9, and F12 was 250.37 ± 23.47, 125.07 ± 12.56, and 247.67 ± 45.79 nm, respectively (Table 2). The particle size of F3 and F9 was larger than that of their corresponding vehicles without PPD, whereas the particle size of F12 was slightly lower. For the effect of pH and osmolarity on the dispersibility of formulation, F3, F9 and F12 were dispersed in simulated gastric fluid (pH 1.2 and 433.3 mOsm/L) and intestinal fluid (pH 6.8 buffer and 77.8 mOsm/L). Both buffers were prepared with reference to the current United States Pharmacopeia (USP). The particle sizes at pH 1.2 and pH 6.8 were 268.47 ± 34.53 for F3 and 256.70 ± 12.65 nm, 112.87 ± 11.96 for F9 and 118.37 ± 2.83 nm, 270.83 ± 33.75 and 265.93 ± 52.14 nm for F12, respectively. There was no significant difference in particle size between pH 1.2 and pH 6.8 buffer. Furthermore, the particle sizes were similar to the dispersion of water as shown at Table 2. Thus, these formulations were consistently dispersed regardless of medium pH and ionic strength.

### 3.4. SEM Analysis of Formulations

**Figure 5:**
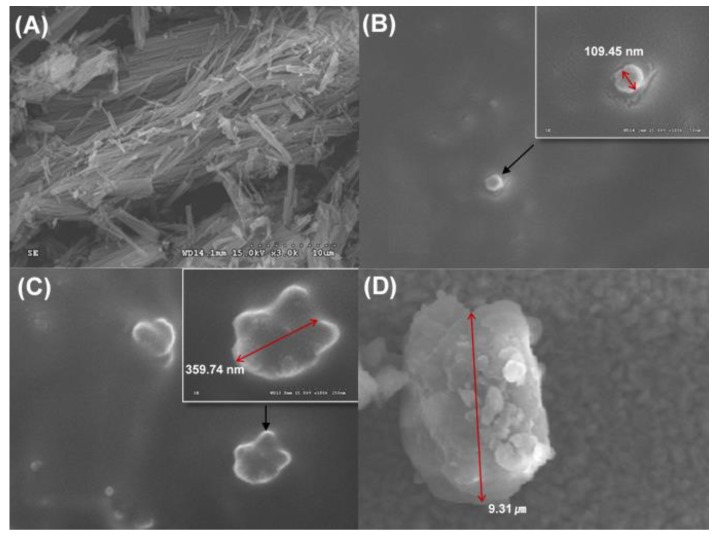
SEM images of raw PPD (**A**), a dispersion particle of F9 (**B**) and F12 (**C**), and a precipitation particle of F4 (**D**).

The morphology of raw PPD or the PSP formulations dispersed in water was examined by SEM. SEM images showed that raw PPD had a needle-shaped form with a particle size of more than 10 µm (Figure 5A). In F9 and F12, the spherical nanoemulsion droplet was observed (Figure 5B,C), and the particle sizes were determined to be 109.45 nm and 359.74 nm, respectively. Particle size values obtained by DLS were confirmed by SEM data for the PSP formulations. However, the results revealed that F4 showed precipitation with a particle size of 9.31 µm, much larger than particles from F9 and F12 (Figure 5D).

### 3.5. Dissolution Test

**Figure 6:**
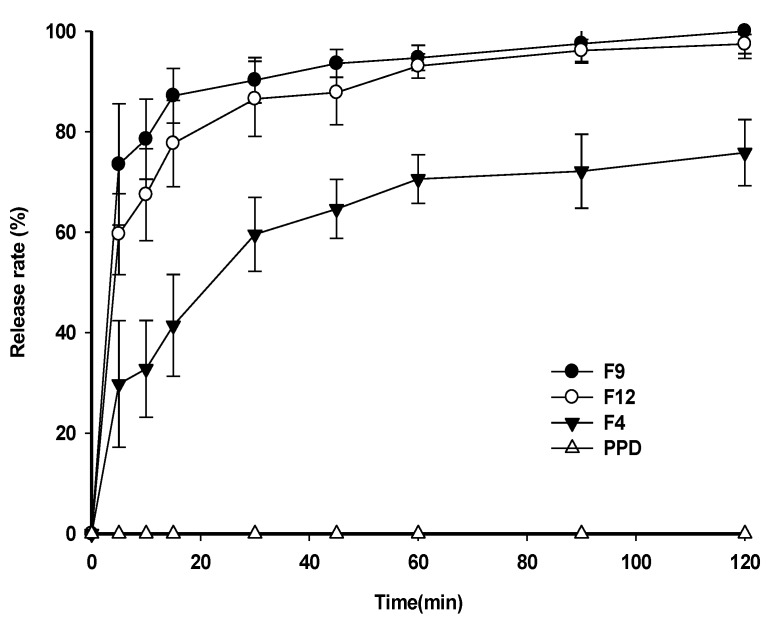
Dissolution profiles of PSP formulations in pH 1.2 buffer.

Dissolution studies were performed using a pH 1.2 buffer at 37 ± 0.5 °C. The dissolution profiles of raw PPD and PSP formulations, such as F4, F9, and F12, are shown in Figure 6. In the case of raw PPD, the significant dissolution of raw PPD was not quantitively detectable by HPLC. The dissolution rate of raw PPD could not be estimated owing to the low aqueous solubility of PPD. In contrast, F9 and F12 showed rapid dissolution rates, reaching 87.15 ± 5.45% and 77.65 ± 8.59%, respectively, within 15 min. The slightly higher dissolution rate of F9 compared to that of F12 appeared to be caused by the mean particle size difference (125.07 ± 12.56 nm for F9; 247.67 ± 45.79 nm for F12). F4 showed a low dissolution rate of 41.45 ± 10.11% in 15 min, which might be related to the formation of PPD precipitates.

### 3.6. Oral Pharmacokinetic Study in Rats

**Figure 7:**
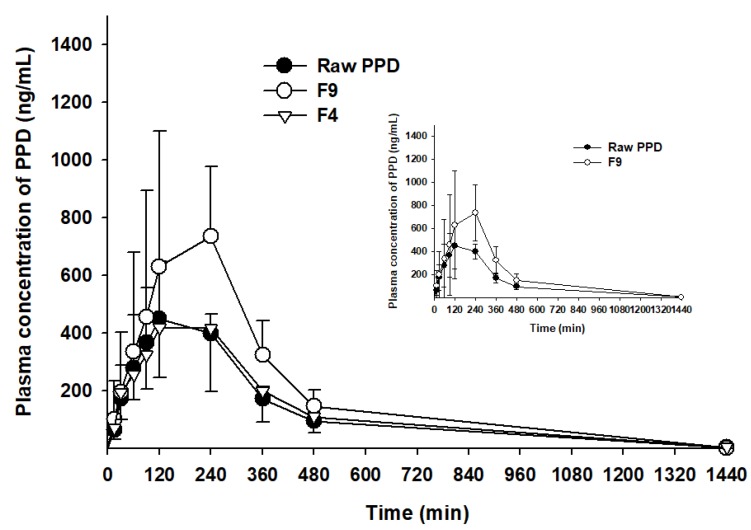
Plasma concentration–time profiles of PPD in Sprague Dawley rats after oral administration of raw PPD, F4 and F9 at a dose of 10 mg/kg (*n* = 4).

As F9 showed the highest dissolution rate among the PSP formulations (Figure 6), it was further compared to F4 or raw PPD in in-vivo oral pharmacokinetic studies. The plasma-concentration-versus-time profiles are shown in Figure 7. The results showed that the plasma concentration of F9 was higher than that of F4 or raw PPD in suspension. In detail, both the C_max_ and AUC_inf_ values of the formulation were significantly higher (1.94- and 1.81-fold, respectively) than those of raw PPD, as shown in Table 3 (*p* < 0.05). In contrast, F4 failed to enhance oral systemic exposure such as C_max_ and AUC_inf_ values compared to raw PPD in suspension (*p* > 0.05, Table 3). Finally, the relative BA of F9 was 181%, strongly indicating that the PSP formulation (F9) enhanced the oral absorption of PPD in vivo, consistent with the dissolution profile data (Figure 6).

## 4. Discussion

Insufficient oral bioavailability of drugs caused by poor aqueous solubility and slow dissolution rate under physiological conditions has been an important issue and should be resolved during the drug development process to improve the clinical success of the drug. SNEDDS has received much attention in recent years as a promising formulation strategy to increase the solubility and dissolution rate of poorly water-soluble drugs and ensure enhanced bioavailability. However, there are limitations in determining the optimal composition of SNEDDS formulations based on the conventional phase diagram constructed using oil, surfactant, and water [17,19]. The previously used phase diagrams require the preparation and evaluation of a large number of preparations. To develop a novel SNEDDS in a preconcentrate state with improved physicochemical stability, we herein suggest a new approach using a modified phase diagram without the water component. In the present study, this comprehensive method was applied to develop novel SNEDDS formulations loaded with PPD, an aglycone of ginsenoside with pharmacologically beneficial effects, to determine the optimal compositions of the vehicle system.

Previous studies have reported that drug delivery systems containing micro- or nano-sized ginsenoside materials improve the in-vivo bioavailability of drugs [23,24,25]. In this study, various types of oils and surfactants were evaluated to prepare PSP formulations with stable and uniform particle sizes. First, PPD solubility in each type of oil or surfactant was evaluated as it is preferable to use vehicle components possessing sufficient ability to solubilize PPD to prepare a PSP. The difference in PPD solubility between two temperature 30 °C and 50 °C was not considerable in either oil or surfactant, but semi-solid and solid materials were more easily agitated at 50 °C. Campul MCM C8 and Capryol 90 have structures similar to oil substances derived from caprylic acid. PPD solubility in these oil-types was above 20% (w/w), much higher than that in Kollisolv MCT 70. Thus, Capmul MCM C8 and Capryol 90 were selected as the oil components for PSP formulations. The high solubility of PPD in the selected oils would be likely due to the structural high affinity between hydrocarbon chain from oil and tetracyclic terpene from PPD. There was no significant difference in the enhanced solubility of PPD between vehicles containing surfactant only, Kolliphor EL, Kolliphor PS80 or Kolliphor HS15. Presumably, PPD could not be solubilized using vehicles containing surfactant only due to the hydrophobic ginsenoside structure of PPD. Consequently, the three surfactants were used to prepare vehicles by combining them individually with either of the two selected oils to generate candidate PSP formulations.

Next, to prepare vehicles containing various ratios of oil to surfactant, phase separation and particle size of the vehicle in the dispersion were evaluated because they are essential factors affecting drug dissolution from PSP formulations in aqueous medium [26]. Similar to the finding of a previous report [27], as the surfactant content increased, the phase separation of the vehicle was alleviated, and the mean particle size tended to decrease. Capryol 90 and Kolliphor EL, however, showed the smallest mean particle size (91.63 ± 1.45 nm) when the amount of oil was greater than that of the surfactant (oil:surfactant ratio, 6:4). Only in that case was the particle size smaller than 200 nm, which is the mean particle size of general types of SNEDDS [28].

Ultimately, based on the results of the solubility test and physical characterization of the vehicles, a ternary phase diagram comprising oils, surfactants, and PPD was constructed to generate candidate PSP formulations. In previous reports, phase diagrams have been used to indicate the solubilization regions of drugs and generate SNEDDS compositions using the physical state and particle size of the mixture. [16,23]. This conventional method requires numerous tests for the physical characterization of vehicles containing various ratios of different types of oils and surfactants. Therefore, it is a labor-intensive approach. In the present study, however, a comprehensive phase diagram was created using the particle size of the drug-free vehicle compositions, and the calculated solubility curve of the drug in each vehicle combination was used to fabricate SNEDDS candidates with optimal composition. This method was more efficient than the conventional method, because only a few selected formulations needed to be evaluated for particulate-related properties, such as particle size and physical state. Twelve formulations were selected for further evaluation based on this comprehensive phase diagram approach.

The purpose of devising a PSP system is to disperse a drug uniformly when a formulation is exposed to water after oral administration via the spontaneous formation of an emulsion in the gastrointestinal tract for facilitated absorption [29]. Therefore, precipitation or phase separation in an aqueous medium should be avoided. When the candidate formulations were dispersed in water, the particle sizes tended to be higher than those of the vehicles only. Specifically, in the PSP formulation comprising Capryol 90 and Kolliphor EL, precipitation occurred within a short time (2 h), when the oil content was less than 60% of the total weight, which was probably caused by reduction in the oil content that facilitated PPD solubilization in the system. In addition, precipitation occurred in the formulations with a PPD content (%) close to the boundary compositions of the soluble region. Through validation of the formulation compositions, the PPD-soluble region of the ternary phase diagram was confirmed to be appropriate for the prediction of the actual solubility of the PSP formulations in aqueous medium.

Among the evaluated formulations, F9 was chosen as the final PSP formulation because it showed no precipitation of PPD with an appropriate particle size distribution when dispersed in water. In addition, in a preliminary stability test, F9 was physicochemically stable during at least 6 months at room temperature (data not shown). In the dissolution test, we investigated whether the particle size of the PSP formulation affects its dissolution rate in an aqueous buffer. As the particle size decreased, the surface area increased, leading to a faster initial dissolution rate [30]. For example, F9 showed higher dissolution rates than F12 within 10 min (Figure 6). The dissolution rate of F4, which showed precipitation in water, was approximately 20% lower than that of F9 and F12 at 60 min.

In the in-vivo oral pharmacokinetic studies, F9 showed higher plasma PPD concentration than raw PPD with a relative BA of 181%. A recent study has reported formulations of novel dry suspension (1446–1653 nm) and dry emulsion (652.8–784.5 nm) of PPD, demonstrating dramatically enhanced aqueous solubility and higher perfusion of PPD in rat intestines. However, they failed to enhance the oral bioavailability in terms of AUC and C_max_ [31]. Moreover, F4 containing PPD precipitation with a particle size of 9.31 µm, much larger than particles from F9 (Figure 4 and Figure 5), could not enhance the oral systemic exposure of PPD, compared to raw PPD in suspension (Table 3). Despite a limited case, we speculated that the successfully enhanced oral absorption of F9 observed in this study was likely due to the formation of smaller nano-sized particles (125 nm).

## 5. Conclusions

A comprehensive ternary phase diagram, as an effective tool, successfully enables the design of optimized formulations with minimal experimentation to measure the solubility and particle size of each vehicle composition. This study demonstrated that, based on the systemized strategy, it was possible to develop an optimal SNEDDS formulation containing the lipophilic compound PPD, an aglycone of ginsenoside with pharmacologically beneficial effects, which improved the oral bioavailability of the drug by forming uniform nano-sized particles and enhancing dissolution upon oral administration.

## Figures and Tables

**Table 1:** Particle size and dispersibility of the vehicle in water.

Oil	Surfactant	Weight Ratio	Particle Size (nm)	PDI
Capmul MCM C8	Kolliphor EL	8:2	Phase separation	N.D.
		6:4	Phase separation	N.D.
		5:5	372.67 ± 45.39	0.28 ± 0.01
		4:6	198.01 ± 14.24	0.19 ± 0.02
		2:8	Clear solution	N.D.
	Kolliphor PS80	8:2	Phase separation	N.D.
		6:4	Phase separation	N.D.
		5:5	352.87 ± 28.07	0.34 ± 0.02
		4:6	277.93 ± 38.30	0.23 ± 0.03
		2:8	164.70 ± 3.83	0.37 ± 0.01
	Kolliphor HS15	8:2	Phase separation	N.D.
		6:4	Phase separation	N.D.
		5:5	Phase separation	N.D.
		4:6	323.23 ± 40.50	0.27 ± 0.03
		2:8	Clear solution	N.D.
Capryol 90	Kolliphor EL	8:2	Phase separation	N.D.
		6:4	91.63 ± 1.45	0.24 ± 0.01
		5:5	195.67 ± 14.32	0.39 ± 0.01
		4:6	277.00 ± 45.81	0.30 ± 0.03
		2:8	115.97 ± 8.57	0.40 ± 0.02
	Kolliphor PS80	8:2	Phase separation	N.D.
		6:4	Phase separation	N.D.
		5:5	Phase separation	N.D.
		4:6	389.70 ± 7.40	0.29 ± 0.03
		2:8	197.40 ± 57.60	0.26 ± 0.01
	Kolliphor HS15	8:2	Phase separation	N.D.
		6:4	Phase separation	N.D.
		5:5	Phase separation	N.D.
		4:6	270.57 ± 24.79	0.32 ± 0.02
		2:8	182.90 ± 17.56	0.23 ± 0.01

N.D., Not detected; PDI, polydispersity index.

**Table 2:** Weight compositions and particle sizes of PSP formulations.

Formulation	PPD	Oil	Surfactant	Particle Size (nm)	PDI
		Capmul MCM C8	Kolliphor EL		
F1	5	19	76	Precipitation	N.D.
F2	10	36	54	Precipitation	N.D.
		Capmul MCM C8	Kolliphor PS80		
F3	5	19	76	250.37 ± 23.47	0.26 ± 0.01
F4	10	36	54	Precipitation	N.D.
		Capmul MCM C8	Kolliphor HS15		
F5	5	19	76	Precipitation	N.D.
		Capryol 90	Kolliphor EL		
F6	5	19	76	Precipitation	N.D.
F7	10	36	54	Precipitation	N.D.
F8	10	45	45	Precipitation	N.D.
F9	10	54	36	125.07 ± 12.56	0.23 ± 0.01
		Capryol 90	Kolliphor PS80		
F10	5	19	76	Precipitation	N.D.
		Capryol 90	Kolliphor HS15		
F11	5	19	76	Precipitation	N.D.
F12	10	36	54	247.67 ± 45.79	0.21 ± 0.03

PDI, polydispersity index; N.D., Not detected.

**Table 3:** Pharmacokinetic parameters of PPD in male rats after oral administration of an equivalent dose (10 mg/kg) of raw PPD and F9 (*n* = 4).

Pharmacokinetic Parameters	Raw PPD in Suspension	PSP (F4)	PSP (F9)
T_max_ (min)	150 ± 60	150 ± 60	160 ± 69
C_max_ (μg/mL)	0.486 ± 0.132	0.453 ± 0.180	0.942 ± 0.206 *
t_1/2_ (min)	197 ± 53	170 ± 46	169 ± 6
AUC_last_ (μg·min/mL)	179 ± 37	186 ± 88	324 ± 83 *
AUC_inf_ (μg·min/mL)	180 ± 37	188 ± 86	325 ± 83 *
MRT (min)	301 ± 41	291 ± 63	281 ± 49
Relative BA (%)		104	181

* *p* < 0.05, compared with raw PPD in suspension.
